# A Comprehensive Nonlinear Multiaxial Life Prediction Model

**DOI:** 10.3390/ma18174185

**Published:** 2025-09-05

**Authors:** Zegang Tian, Yongbao Liu, Ge Xia, Xing He

**Affiliations:** College of Power Engineering, Naval University of Engineering, Wuhan 430033, China; 18835219305@139.com (Z.T.); hexing_mail@163.com (X.H.)

**Keywords:** stress gradient, additional hardening, life prediction, numerical simulation

## Abstract

Compressor blades are subjected to multiaxial loads during operation. Using uniaxial life prediction formulas to predict their fatigue life can result in significant errors. Therefore, by analyzing the loading conditions of the blades, a fatigue life prediction model suitable for compressor blades was developed. This model was established by applying the load of a specific engine type to a notched bar specimen and considering the gradient and strengthening effects. Firstly, the parameters of the SWT model were used as the damage parameters to determine the critical plane location based on the principle of coordinate transformation, and these results were compared with the actual fracture angles. Additionally, the physical mechanisms of multiaxial fatigue crack initiation and propagation were investigated at the microscopic level. Secondly, the non-uniform stress field on the critical plane was obtained using the finite element method. The stress distribution from the critical point to the specimen’s principal axis was extracted and normalized to calculate the equivalent stress gradient factor. Finally, the results of the comprehensive fatigue life prediction model were computed. Comparisons between the calculated results of the proposed model, the SWT model, and the Shang model with the experimental fatigue life showed that the prediction accuracy of the proposed model is higher than that of the SWT model and the Shang Deguang model.

## 1. Introduction

Gas turbines, with their high power density and fast start-up capabilities, have been widely used in various industries. The innovative development of gas turbines has imposed comprehensive requirements on compressors, such as high flow rate, high pressure ratio, and high thermal efficiency, which in turn pose greater challenges to the reliability of compressor blade service. The main failure modes of compressor blades are foreign object damage, deformation elongation, and fatigue fracture. According to statistics from the AVIC (Aviation Industry Corporation of China), 81% of the failure modes of faulty blades are various forms of fatigue cracks or fatigue fractures [1]. Since compressor blades often endure complex loading conditions formed by the combined action of centrifugal loads, unsteady aerodynamic loads, and alternating mechanical loads during service, multiaxial fatigue failure is a major form of failure for gas turbine compressor blades. To enhance the reliability of blade usage and reduce accidents caused by blade fatigue failure, it is essential to fully understand the damage accumulation laws of blade materials under multiaxial loading. In recent years, numerous scholars have proposed different viewpoints on experimental and research methods for blade multiaxial fatigue. Regarding experimental methods, the primary approaches involve using new-generation electrohydraulic servo testing machines and different types of specimens. For example, Liu et al. [2] validated the yield criterion of aluminum alloy materials using cruciform tensile specimens and found that it could describe the yielding process well. Veronique Duquette, H. Nakamura et al. [3,4] conducted experiments on thin-walled titanium alloy tubular specimens under tensile–torsional multiaxial non-proportional loading to investigate the fatigue crack initiation and propagation behavior of the material under such loading conditions. Currently, the primary approach for studying the multiaxial fatigue of blades involves integrating finite element analysis with critical multiaxial fatigue testing for validation. Zhao et al. [5] conducted a stress analysis on the compressor of a certain type of dual-rotor system engine using ANSYS 2016. They found that under aerodynamic loading alone, the maximum stress of the blades was located at their roots. Under combined aerodynamic and centrifugal loading, the critical stress of the low-pressure compressor blades was located at their roots, while that of the high-pressure compressor was located at the disk roots. The blade root refers to the connection between the blade body and the tenon. Therefore, according to the stress characteristics, both locations can be classified as notched components. Due to geometric discontinuities in the blade, the stress state in local regions becomes complex, making the blade more susceptible to crack initiation and fracture. As research on stress concentration has deepened [6], it has been found that stress gradually decreases from the surface to the interior in regions where stress is concentrated, thereby generating a stress gradient effect. Studies have shown that the impact of the stress gradient on the assessment of fatigue life and fatigue strength is significant. In summary, notched components should be used when exploring the damage accumulation laws of TC4 blade materials under multiaxial loading. Domestic and foreign scholars have conducted relevant research on the multiaxis life prediction of defective components [7,8,9].

**Figure 1:**
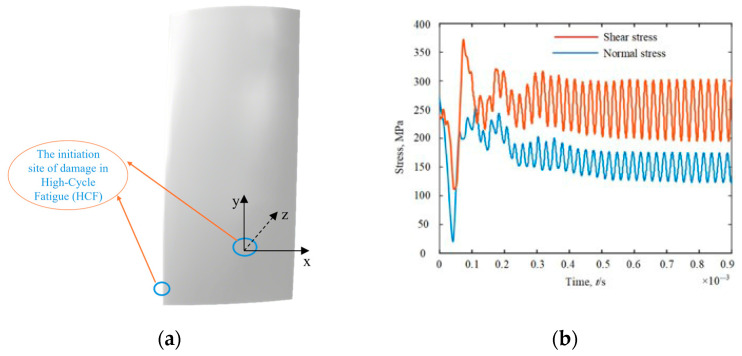
Locations of critical points and stress conditions for blades’ high-cycle fatigue [10]. (**a**) Schematic diagram of the HCF failure location of the rotor blade. (**b**) Variation in σx and τxy at the HCF failure location with time.

During the service life of gas turbines, Li et al. [10] found through numerical simulation that the normal stress σ_x_ and shear stress τ_xy_ at the initiation site of high-cycle fatigue (HCF) damage on the compressor blades have the same frequency but a certain phase difference, and they are not completely proportional to loading, as shown in Figure 1.

**Figure 2:**
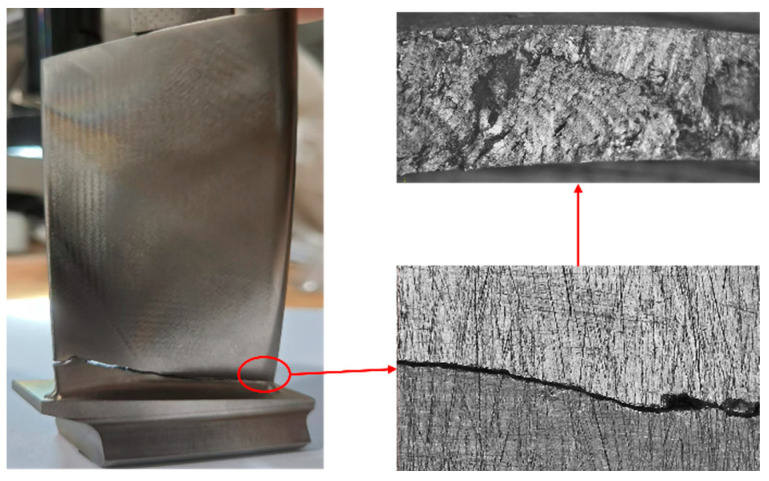
Fracture surface condition of the 8th-stage compressor blade of a certain type of gas turbine.

**Figure 3:**
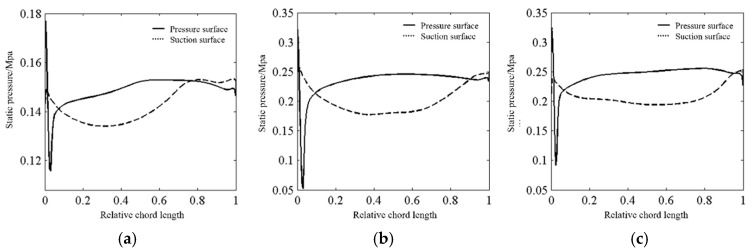
Static pressure distribution on the blade surface at different blade heights [11] (**a**) 15% blade height; (**b**) 50% blade height; (**c**) 85% blade height.

Under non-proportional loading, materials are prone to additional hardening effects. During operation, the dominant orientations of stress and strain in the blades are subject to continuous variation, which impedes the generation of stable dislocation structures within the material, thereby causing more microcracks to occur inside the material, as shown in Figure 2. During crack propagation, the increase in the number of cracks accelerates the crack growth rate, thereby reducing the fatigue life of the blades. When the loading of the blades is non-proportional, the cyclic constitutive relationship characterizing the material’s response to stress and strain becomes complex, which also makes the fatigue life prediction or strength verification of the blades under multiaxial non-proportional loading more difficult. Li et al. [11] used a fluid–structure interaction method to analyze the stress distribution at different blade heights of a certain type of gas turbine compressor blade and found that in the non-stall state, the pressure on the pressure side of the compressor blade is greater than that on the suction side. The periodic aerodynamic load acting on the blade is always greater than zero, and the periodic load acting on the blade is asymmetric. The variation in the static pressure distribution on the moving blade surface at different blade heights is shown in Figure 3. Under asymmetric loading, the maximum stress area of the cyclic load causing material fatigue failure is relatively higher. The blade material undergoes local plastic deformation under higher stress, which in turn changes the fatigue damage accumulation law.

Therefore, this paper establishes a multiaxial fatigue damage life prediction model suitable for predicting blade life, based on damage mechanics and considering the effects of stress gradient and additional hardening due to non-proportional loading on multiaxial fatigue life. First, this paper analyzes the nonlinear life prediction model and finds that the lifetime is mainly influenced by the stress amplitude $\frac{\Delta \sigma }{2}$. Finite element analysis and Matlab 2021a software were used to calculate the SWT parameter, and the critical angle corresponding to the maximum SWT parameter is the location of the critical plane. The stress profile extending from the critical point to the center point on the critical plane is normalized to determine the stress gradient. Combining Borodii’s definition of the non-proportional factor, a multiaxial life prediction model suitable for compressor blades is proposed. The model considers additional hardening and stress gradient effects during a blade’s service life, making it more consistent with the actual working conditions of compressor blades. Finally, fatigue tests were conducted on the TC4 material commonly used for blades, and the causes of additional hardening and the main fracture mechanisms were described from a microscopic perspective. The proposed model was compared with the commonly used SWT model and the nonlinear fatigue life estimation model proposed by Shang Deguang (hereinafter referred to as the SDG model) to verify its accuracy.

## 2. Nonlinear Estimation Model

The problem of material damage has the following characteristics: there are different stages of crack initiation and propagation, nonlinear cumulative effects exist under multilevel loading, damage significantly decreases after reaching the fatigue limit, there are effects from the mean stress, etc. The nonlinear damage accumulation model proposed by Chaboche et al. [12], based on continuum damage mechanics and considering the above characteristics, has been extensively employed to address issues regarding the fatigue life of components, as shown in Equation (1). The function f is required to be inseparable from the loading parameters and damage variables in order to better describe nonlinear damage accumulation and the sequence effects of loading [13].

(1) dD=f(D,σ)dN

When the loading form is stress and it is single-axis fatigue, Chaboche et al. [12], based on Equation (1), formulated the damage accumulation expression shown in Equation (2), which can evolve into

(2) $$\frac{dD}{dN}={\left[1-D\right]}^{\alpha }{\left(\frac{\sigma_{\max}-\sigma_{m}}{M_{0}(1-b^{\prime }\sigma_{m})}\right)}^{\beta }$$

(3) $$\alpha =\left\{\begin{array}{cc} 1-a_{1}\frac{\sigma_{\max}-\sigma_{l}(\sigma_{m})}{\sigma_{b}-\sigma_{\max}} & ,\frac{\sigma_{\max}-\sigma_{l}(\sigma_{m})}{\sigma_{b}-\sigma_{\max}}>0\\ 1 & ,\frac{\sigma_{\max}-\sigma_{l}(\sigma_{m})}{\sigma_{b}-\sigma_{\max}}<0 \end{array}\right.$$

(4) $$\sigma_{l}(\sigma_{m})=\sigma_{m}+\sigma_{-1}(1-b_{0}\sigma_{m})$$

In this equation, D is the damage variable; N represents the lifespan variable; $M_{0}$, $b^{\prime }$, β, and $a_{1}$ are material parameters; $\sigma_{\max}$ is the maximum stress of cyclic loading; $\sigma_{b}$ is the tensile strength of the material; $\sigma_{l}(\sigma_{m})$ is the fatigue limit under asymmetric loading, which is related to the mean stress $\sigma_{m}$; and $\sigma_{-1}$ is the fatigue limit under symmetric cyclic loading (with a stress ratio R of −1).

It is commonly believed that when the blade is in the initial undamaged state, D is 0, and when the crack propagates to the specified length of a macroscopic crack, D is 1. Therefore, by integrating D over [0, 1], we can obtain the fatigue life $N_{f}$ [14], as shown in Equation (5).

(5) $$N_{f}=\frac{1}{1-\alpha }{\left(\frac{\sigma_{\max}-\sigma_{m}}{M_{0}(1-b^{\prime }\sigma_{m})}\right)}^{-\beta }$$

Substituting the stress amplitude into Equation (5) yields

(6) $$N_{f}=\frac{{\left[M_{0}(1-b^{\prime }\sigma_{m})\right]}^{\beta }}{1-\alpha }{\left(\frac{\Delta \sigma }{2}\right)}^{-\beta }$$

From Equation (6), it is evident that the prediction outcomes of the uniaxial fatigue life prediction model are predominantly influenced by $\frac{\Delta \sigma }{2}$. Therefore, the multiaxial fatigue damage model established based on the uniaxial fatigue damage model mainly differs in terms of the definition of the control variable $\frac{\Delta \sigma }{2}$. Shang et al. [15] developed a multiaxial fatigue model that employs the equivalent stress amplitude $\frac{\Delta \sigma_{\mathrm{eq}}}{2}$ to replace the control variable $\frac{\Delta \sigma }{2}$ in the uniaxial fatigue damage model, but due to the neglect of stress concentration and additional hardening effects, the accuracy of predicting the service life of components with geometric discontinuities, such as blades, is not high.

## 3. Notch Stress Gradient Factor

Findley [16] first introduced the notion of the critical plane to investigate multiaxial fatigue. Assuming that crack initiation commences on the critical plane. The multiaxial loads are projected onto possible planes, the fatigue damage parameters on each plane are calculated, and the plane with the highest damage parameter is identified as the critical plane, which provides a basis for predicting blade crack locations. Głowacka K et al. [17] also conducted multiaxis fatigue analysis for some key planes of composite laminates. Arora P et al. [18] used the interface method to make predictions for 17 types of materials. Smith et al. believed that component fatigue failure is primarily determined by the normal strain amplitude $\frac{\Delta \varepsilon_{n}}{2}$ and the maximum normal stress $\sigma_{n,\max}$ on the critical plane, as shown in Equation (7), where $\sigma_{f}^{\prime }$ represents the fatigue strength coefficient, $\varepsilon_{f}^{\prime }$ represents the fatigue ductility coefficient, and b and c, respectively, denote the fatigue strength index and the fatigue ductility index. This effectively characterizes the impact of the maximum tensile stress and strain amplitude on multiaxial fatigue life.

(7) $$\sigma_{n,\max}\frac{\Delta \varepsilon_{n}}{2}=\frac{\sigma_{f}^{\prime 2}}{E}{\left(2N_{f}\right)}^{2b}+\sigma_{f}^{\prime }\varepsilon_{f}^{\prime }{\left(2N_{f}\right)}^{b+c}$$

Therefore, based on the principle of coordinate transformation, the states of points σ and ε on the dangerous inner part of the notch component can be expressed as

(8) $$\sigma_{ij}=\left[\begin{array}{ccc} \sigma_{11} & \tau_{12} & \tau_{13}\\ \tau_{21} & \sigma_{22} & \tau_{23}\\ \tau_{31} & \tau_{32} & \sigma_{33} \end{array}\right],\varepsilon_{ij}=\left[\begin{array}{ccc} \varepsilon_{11} & \varepsilon_{12} & \varepsilon_{13}\\ \varepsilon_{21} & \varepsilon_{22} & \varepsilon_{23}\\ \varepsilon_{31} & \varepsilon_{32} & \varepsilon_{33} \end{array}\right]$$

**Figure 4:**
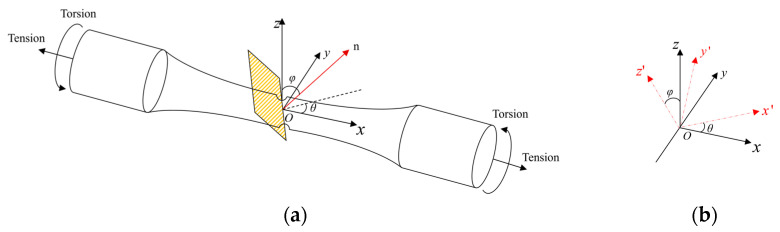
(**a**) Critical plane location and orientation. (**b**) Diagram of coordinate system transformation.

The critical plane’s location and orientation are shown in Figure 4a, where the angle between the normal vector n of the plane and the positive direction of the z-axis is denoted as φ and the angle between the projection of n on the XOY plane and the x-axis is denoted as θ. The plane OXY is rotated by an angle θ to obtain the plane OX’Y’, and the Z-axis is rotated by an angle φ around the X’ axis to obtain the coordinate axis Z’. The coordinates x’y’z’ represent the transformed coordinate system. The stress and strain at any θ and φ can be calculated through the rotation matrix M:

(9) $$\begin{array}{l} \varepsilon_{ij}^{\prime }=M\varepsilon_{ij}M^{T}\\ \sigma_{ij}^{\prime }=M\sigma_{ij}M^{T} \end{array}$$

The rotation matrix M can be expressed as

(10) $$M=\left[\begin{array}{ccc} \cos\theta \sin\phi & \sin\theta \sin\phi & \cos\phi \\ -\sin\theta & \cos\theta & 0\\ -\cos\theta \cos\phi & -\sin\theta \cos\phi & \sin\phi \end{array}\right]$$

Therefore, when the SWT parameter reaches its maximum value, the critical angle can be used to determine the critical plane of the point to be evaluated, and the corresponding SWT parameter can be represented as [19]:

(11) $$\begin{array}{l} \underset{i}{\max}\left\{{\mathrm{SWT}}^{i}\right\}=\underset{i}{\max}\left\{\sigma_{n}^{i}\left(\frac{\Delta \varepsilon_{n}^{i}}{2}\right)\right\}=\underset{1\leq m\leq s}{\max\left|\sigma_{ii}\left(m\right)\right|}\underset{1\leq m\leq s,m+1\leq n\leq s}{\max}\left|\varepsilon_{ii}\left(m\right)-\varepsilon_{ii}\left(n\right)\right|\\ \vec{n}=\left(\begin{array}{c} \sin\phi \cos\theta ,\sin\phi \sin\theta ,\cos\phi \end{array}\right)\\ \begin{array}{c} \sigma_{ii}=\cos^{2}\theta \sin^{2}\phi \sigma_{11}+2\sin\theta \cos\theta \sin^{2}\phi \sigma_{12}+2\cos\theta \sin\phi \cos\phi \sigma_{13}\\ +\sin^{2}\theta \sin^{2}\phi \sigma_{22}+2\sin\theta \sin\phi \cos\phi \sigma_{23}+\cos^{2}\phi \sigma_{33} \end{array}\\ \begin{array}{c} \varepsilon_{ii}^{\prime }=\cos^{2}\theta \sin^{2}\phi \varepsilon_{11}+2\sin\theta \cos\theta \sin^{2}\phi \varepsilon_{12}+2\cos\theta \sin\phi \cos\phi \varepsilon_{13}\\ +\sin^{2}\theta \sin^{2}\phi \varepsilon_{22}+2\sin\theta \sin\phi \cos\phi \varepsilon_{23}+\cos^{2}\phi \varepsilon_{33} \end{array} \end{array}$$

**Figure 5:**
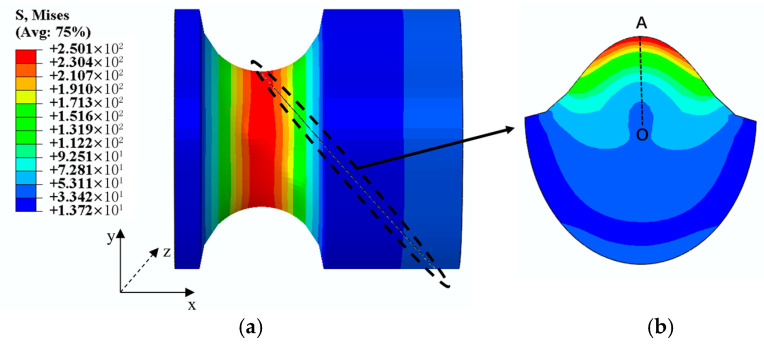
Stress gradient effect and path extraction: (**a**) Determination of the critical plane; (**b**) projection and path of the critical plane on the ZOY plane.

Through numerical simulation, we can observe the stress distribution on the critical plane of the notched specimen, as shown in Figure 5b, which is characterized by a local non-uniform stress distribution, showing a trend of stress decreasing gradually from the notch towards the interior. This effect is known as the stress gradient effect. Numerous studies have found that neglecting the stress gradient effect often leads to conservative predictions of a specimen’s lifetime [20,21,22]. This paper uses the SWT parameter to identify the critical plane, assuming that lifetime is primarily determined by the normal strain amplitude $\frac{\Delta \varepsilon_{n}}{2}$ and the maximum normal stress $\sigma_{n,\max}$ on the critical plane.

Wang Yanrong [23] proposed a stress gradient factor. This was based on uniaxial tension–compression experiments, the normal stress gradient factor $Y_{\sigma }$, and the shear stress gradient factor, $Y_{\tau }$, which are defined as shown in Equation (12).

**Figure 6:**
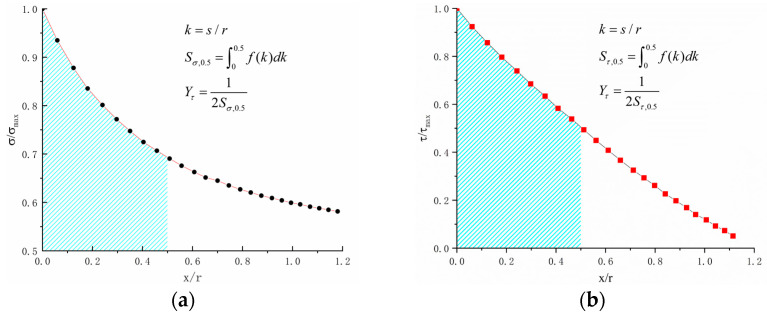
Normalized normal and shear stress distributions: (**a**) normal stress gradient; (**b**) shear stress gradient.

The area enclosed by the curve represented by the normal N along the critical plane path OA within the range C is denoted by $S_{\sigma 0.5}$. In this study, the variable $S_{\sigma 0.5}$ is defined as the area bounded by the normalized normal stress $\sigma /\sigma_{\max}$, as delineated along the critical plane path OA. This is measured within the specified range 0≤x/r≤0.5. The variable $S_{\tau 0.5}$ is representative of the area bounded by the normalized shear stress $\tau /\tau_{\max}$ within the specified range 0≤x/r≤0.5, as shown in Figure 6.

(12) $$\begin{array}{l} Y_{\sigma }=\frac{1}{2S_{\sigma ,0.5}}=\frac{1}{2\int_{0}^{0.5}f(\sigma /\sigma_{\max})d(\sigma /\sigma_{\max})}\\ Y_{\tau }=\frac{1}{2S_{\tau ,0.5}}=\frac{1}{2\int_{0}^{0.5}f(\tau /\tau_{\max})d(\tau /\tau_{\max})} \end{array}$$

In circumstances where a multiaxial load is applied, the normal stress gradient and the shear stress gradient have been shown to exert an influence on the fatigue life of a component. Therefore, the equivalent stress gradients of the normal stress and the shear stress are as follows:

(13) $$Y_{\mathrm{eq}}=\sqrt{Y_{\sigma }Y_{\tau }}.$$

**Figure 7:**
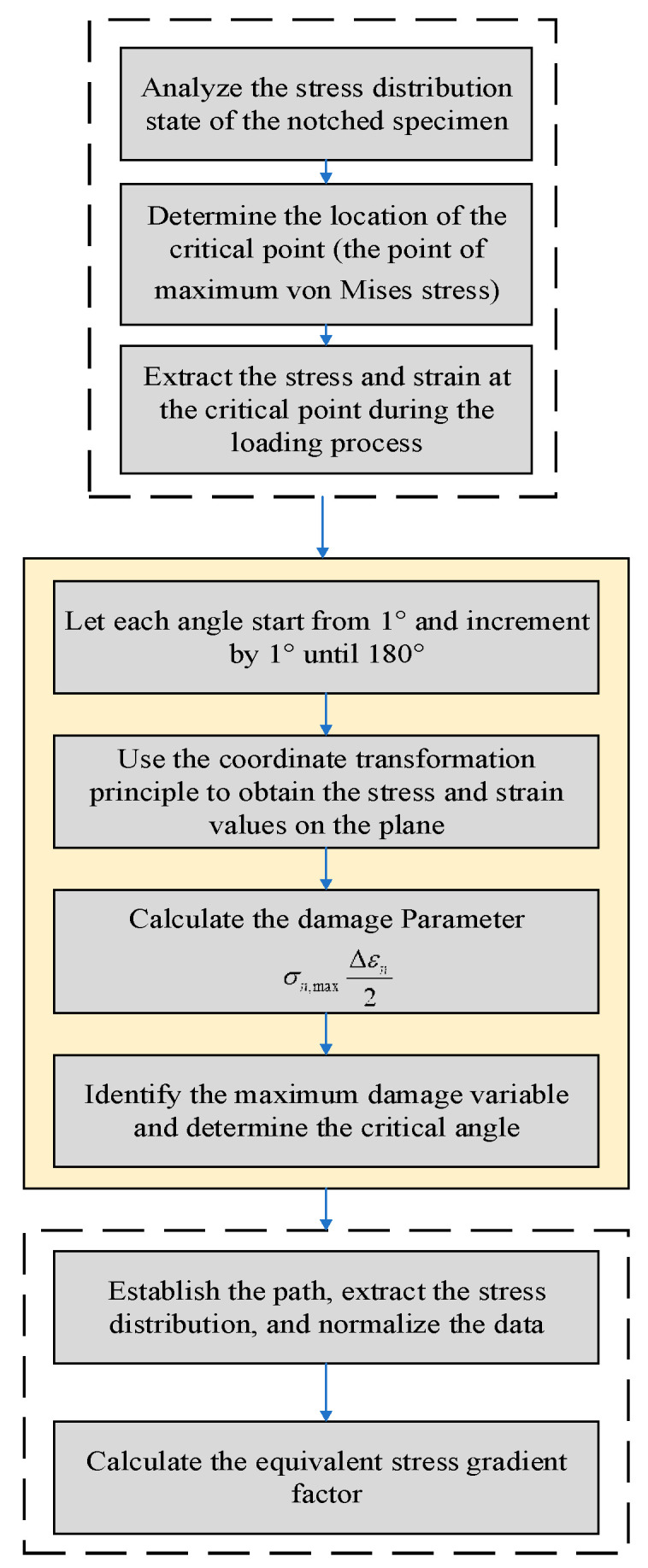
The flowchart for extracting the stress gradient distribution on the critical plane.

The procedure for determining the critical plane location based on the SWT critical plane method is shown in Figure 7: (1) Use ABAQUS to establish the stress distribution of the notched specimen and identify the position of the critical fatigue point. (2) Measure the stress and strain variables at the critical location during the loading procedure, subsequently importing them into the Matlab environment. (3) Let θ and ϕ vary from 1° to 180° in increments of 1°. (4) Use the coordinate transformation principle to obtain the stress and strain coefficients on the aforementioned plane and calculate the damage parameter. (5) Determine the critical plane by identifying the critical angle corresponding to the maximum damage parameter. (6) Establish a straight-line path from the critical point on the critical plane to the center of the specimen section, extract the stress distribution, and normalize it. Figure 6 is the flowchart for extracting the stress gradient distribution on the critical plane.

## 4. Comprehensive Multiaxial Lifetime Prediction Model

**Figure 8:**
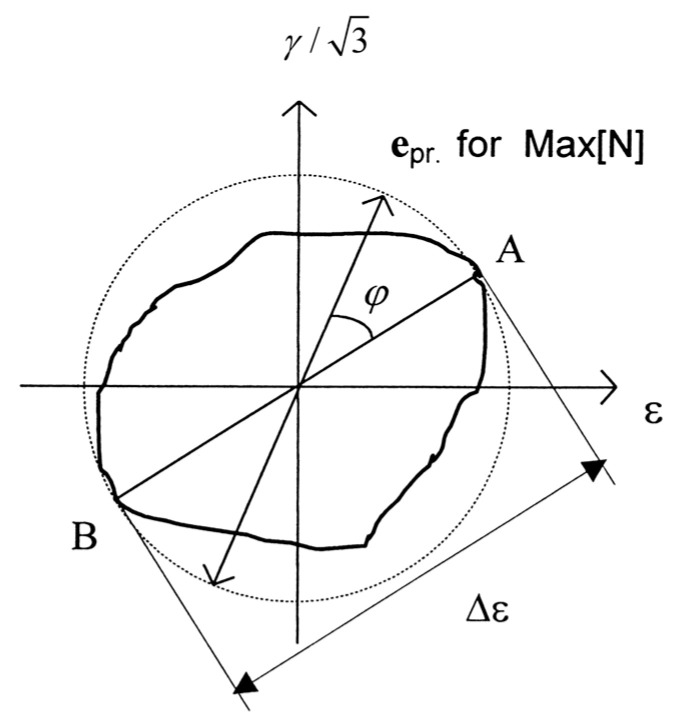
Schematic diagram of the principal directions of an arbitrary cyclic path.

From a microscopic perspective, the additional effect is due to the deviation from proportional loading during stress application, and the formation of stable dislocation structures within the material is made difficult by continuous changes in the principal axes of stress and strain [24]. As shown in Figure 8, path AB represents proportional loading. We believe that the greater the deviation of the non-proportional loading path from the in-phase proportional loading path, the higher the degree of non-proportional additional hardening of the material, and when the maximum strain amplitude is equal for different loading paths, the larger the region enclosed by the equivalent convex path, the greater the degree of additional hardening. When the loading path reaches 90° out-of-phase, the equivalent path forms a circle defined by the maximum strain amplitude and the degree of additional strengthening reaches its maximum.

Therefore, Borodii [25] considered the effect of a shift in the relationship between the strain path and the principal strain axis on fatigue life and proposed a method to describe the degree of deviation from proportional loading for different paths using the non-proportionality factor $l_{\mathrm{np}}$ and the loading non-proportionality degree $f_{\mathrm{np}}$, as shown in Equations (14) and (15).

(14) $$l_{\mathrm{np}}=(1+k\sin\varphi_{a})(1+\alpha f_{np})$$

(15) $$\left\{\begin{array}{l} f_{np}={\left(\left|\begin{array}{c} \oint_{L^{\prime }}\vec{e}\cdot d\vec{e} \end{array}\right|/\left|\begin{array}{c} \oint_{L_{n}}\vec{e}\cdot d\vec{e} \end{array}\right|\right)}^{r},0<f_{np}<1\\ \left.r=(1-\left|\oint_{L}\vec{e}\cdot d\vec{e}\right|/\left|\oint_{L_{0}}\vec{e}\cdot d\vec{e}\right|\right)\frac{l}{4\Delta \varepsilon_{m}}\\ l=\oint_{L}dl \end{array}\right\}$$

**Figure 9:**
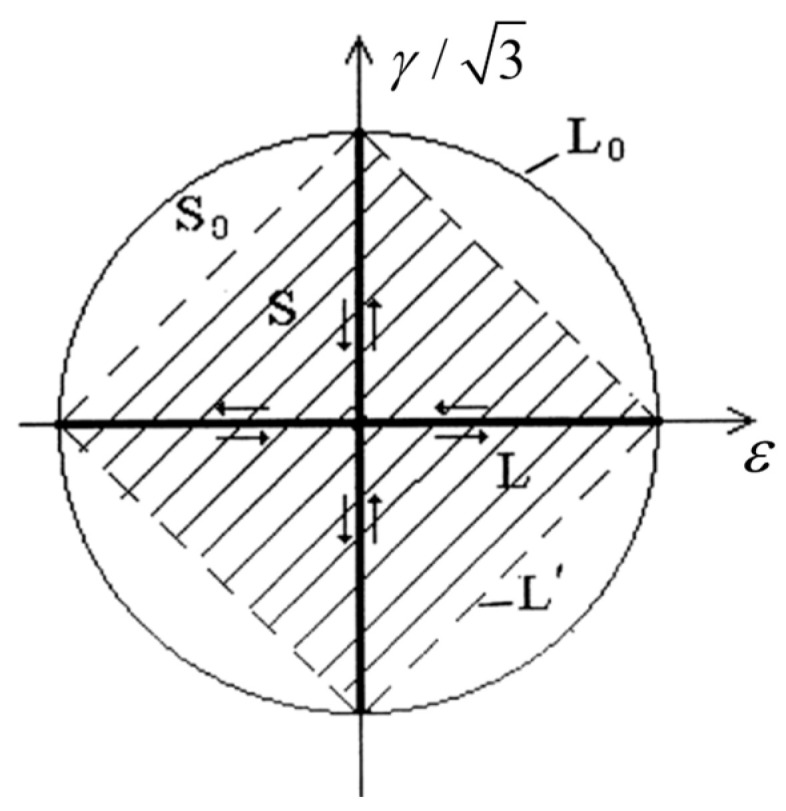
Cyclic path and equivalent convex path.

In this equation, k is a material constant characterizing the difference in the cyclic properties of proportional strain paths; $\varphi_{a}$ is the angle of the cyclic path direction relative to the principal axis; $\vec{e}$ and $d\vec{e}$ are the strain vector and the strain increment vector, respectively; L’ is the equivalent convex path of an arbitrary non-proportional strain path L; and L0 is the maximum circular path of an arbitrary strain path L. For example, for the cruciform loading in Figure 9, the integral of the equation $f_{np}$ is 0, indicating that its path is a non-convex path, and the equivalent convex path L’ should be selected for calculation. Therefore, the equivalent convex path is a piecewise path that includes the actual cyclic path, and the smooth curvature of the equivalent convex path is generally denoted by r.

In conclusion, in Equation (6), the control variable $\frac{\Delta \sigma }{2}$ is modified to the variable $\frac{\Delta \sigma_{np}}{2}$, which takes into account the reinforcing effect. By introducing the equivalent stress gradient factor $Y_{eq}$, a comprehensive multiaxial fatigue life prediction model is ultimately developed, as shown in Equation (16).

(16) $$N_{f}=\frac{{\left[M_{0}(1-b^{\prime }\sigma_{m})\right]}^{\beta }}{1-\alpha }{\left(\frac{\Delta \sigma_{np}}{2}Y_{eq}\right)}^{-\beta }$$

## 5. Simulation and Experimental Validation

**Figure 10:**
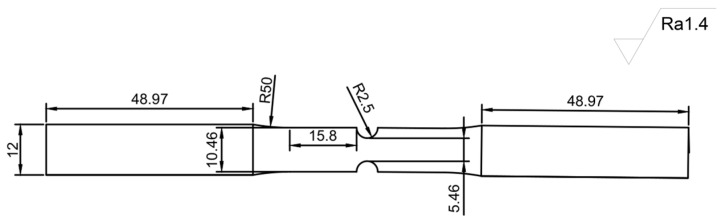
Fatigue specimens of TC4 alloy.

**Figure 11:**
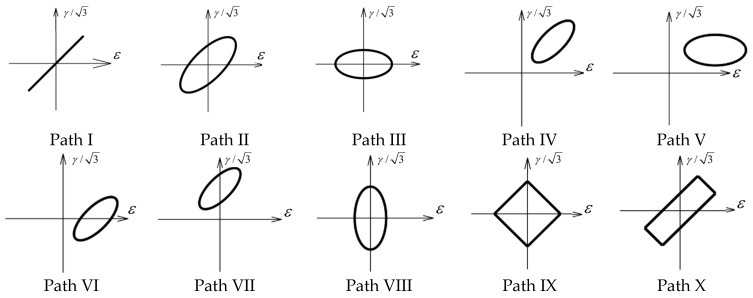
Different loading paths.

The experimental specimens were manufactured by China Anhui Kexun Automation Technology Co., Ltd. (Hefei, China) using a lathe process., with the geometric parameters and material characteristics shown in Figure 10 and Table 1. The chemical composition is shown in Table 2.The multiaxial fatigue tests were conducted by the Institute of Mechanics, Chinese Academy of Sciences, using an MTS809 testing machine (Made by the American MTS Systems Company, with the products provided by its subsidiary in Shanghai, China), with F (kN) and T (N·) as the control variables. The frequency was set at 5 Hz, and the loading path is shown in Figure 11. The test was terminated when the specimen fractured, and the number of cycles at this point was recorded as the fatigue life of the specimen. If the number of cycles reached 10^6^ without fracture, the fatigue limit was not considered to have been reached, and the fatigue life was recorded as 10^6^.

**Figure 12:**
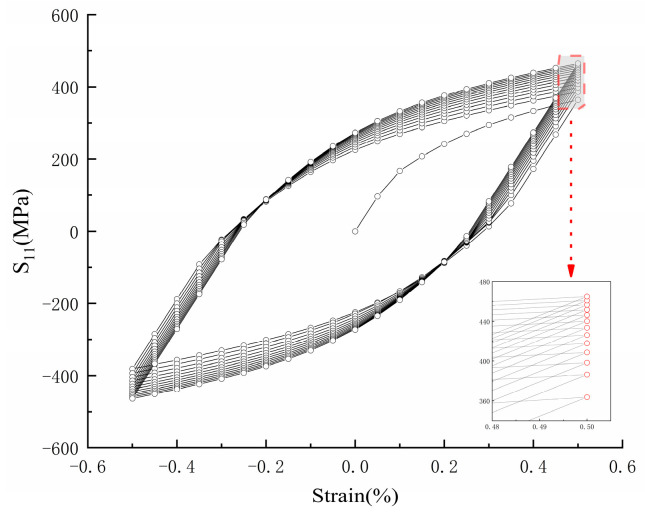
Hysteresis loop curve of TC4.

Finite element simulations were performed using ABAQUS software 6.14, with the material constitutive model set as the nonlinear kinematic hardening Chaboche model, as shown in Equation (17). The constitutive parameters were selected from a study conducted by Wu et al. [26], who found through research that the best description is achieved by using three hardening variables. Thus, *n* was set to 3 for numerical simulation using loading steps to simulate cyclic loading. After multiple cycles, the stress hysteresis loop of the material along the X-axis is shown in Figure 12. The constitutive parameter settings are shown in Table 3.

(17) $$\begin{array}{c} \Delta \alpha_{i}=\frac{2}{3}c_{i}\Delta \varepsilon^{p1}-\gamma_{i}\alpha_{i}\lambda \\ \alpha =\sum_{n}^{1}\alpha_{i} \end{array}$$

In this equation: $c_{i}$ and $\gamma_{i}$ (*i* = 1, 2, …, *n*) are the kinematic hardening material parameters; $\alpha_{i}$ is the hardening variable; and α is the total hardening variable.

**Figure 13:**
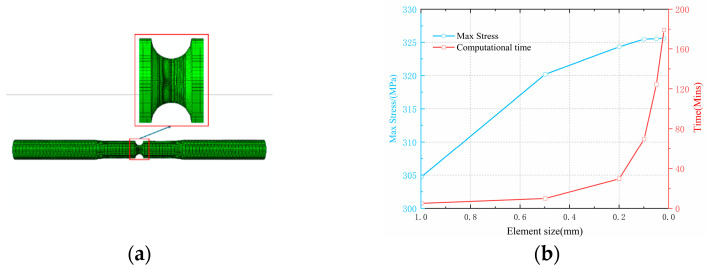
Mesh independence verification and division: (**a**) mesh refinement segment; (**b**) mesh independence verification.

Since the precision of the stress distribution on the notch obtained from finite element calculations directly determines the accuracy of the extracted stress gradient, this paper conducted a mesh independence verification by changing the mesh size at the notch to confirm the validity of the results. Figure 13 illustrates that the maximum stress at the notch diminishes as the mesh size increases, and the stress value stabilizes when the mesh size is below 0.2 mm.

## 6. Results and Discussion

**Figure 14:**
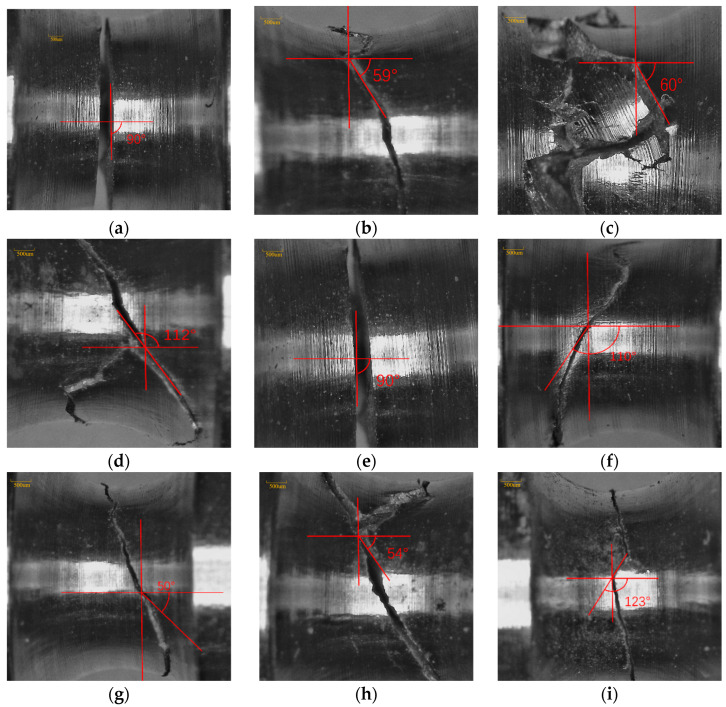
Fracture surfaces of specimens and critical planes calculated by the finite element method: (**a**) 1-I; (**b**) 2-I; (**c**) 3-I; (**d**) 8-II; (**e**) 9-III; (**f**) 10-III; (**g**) 25-I; (**h**) 26-II; (**i**) 27-III.

Table 4 provides the complete experimental data for applied stress amplitude and fatigue life, where, to make the experimental data more targeted, the aerodynamic and centrifugal loads at each stage for the compressor blade of a certain type of engine, provided in [18], are used as the amplitude variations for tension and torsion. Among them, the lifetime data obtained for loading using paths IV and V include two loading cases, where the stress ratios of tensile and torsional loads are 0.7 and 0.1 and the phase difference is 45°, respectively, indicated as (IV-0.1) in the table. The phase difference in paths VI and VII differs among three loading cases, namely 30°, 45°, and 60°. Path VI represents a tensile load stress ratio of −1 and a torsional load stress ratio of 0.1, while path VII represents a tensile load stress ratio of 0.1 and a torsional load stress ratio of −1, indicated as (VI-30°) in the table. Each group of experiments was conducted twice. $\bar{N_{f}}$ represents the average lifetime from the two experiments, and the critical angles and stress gradient factors obtained through finite element analysis and subsequent processing are provided. Figure 14 lists the fracture surface of the experimental specimen and the critical plane calculated by the finite element method. To confirm the validity of the model under diverse loading paths, the model prediction results were validated against paths IX and X in [27].

To verify whether the SWT parameter is suitable for determining the critical plane as the damage parameter, the specimens with serial numbers 1, 2, 3, 8, 9, 10, 25, 26, and 27 were selected to observe and record the fracture surface angles, as shown in Figure 13. It was found that, except for specimens 1, 9, and 26, the initial fracture surface angles $\theta_{d}$ were basically consistent with the calculated critical plane angles ($\frac{\theta_{d}-\theta_{c}}{\theta_{c}}$ < 5%), mainly because the SWT parameter is better suited for tensile crack modes. Meanwhile, for the experimental conditions of specimens 1 and 9, the torsional load was relatively large, and the fracture was more of a torsional crack model, resulting in larger errors when using the SWT model to determine the critical plane for lifetime prediction.

**Figure 15:**
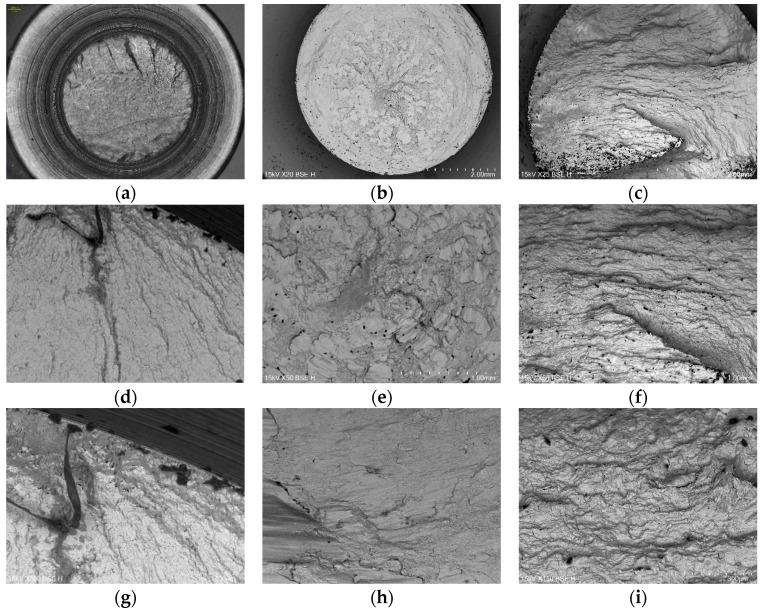
Microscopic analysis of fatigue fracture surfaces. (**a**,**d**,**g**) correspond to the fracture surfaces of specimens under tensile loading only, magnified by 20×, 50×, and 150×; (**b**,**e**,**h**) correspond to the fracture surfaces of specimens under torsional loading only, magnified by 20×, 50×, and 150×; (**c**,**f**,**i**) correspond to the fracture surfaces of specimens under multiaxial loading, magnified by 20×, 50×, and 150×.

Figure 15 provides SEM images of the fracture surfaces of the selected specimens. From a microscopic perspective, under pure tension, distinct radial ridges are observed. The crack propagation bands are initially dense and gradually widen with crack growth. As shown in Figure 15h, under pure shear stress, slip causes material surface extrusion and intrusion; gradually, slip bands form. These slip bands are persistent. They lead to fatigue crack initiation. The crack initiation direction is mostly perpendicular to the specimen axis, which is subjected to cyclic shear stress, indicating a Mode II crack. The scratch marks on the fracture surface confirm this. Under multiaxial non-proportional loading, the principal axes of stress and strain continuously change, which means its fracture mechanism cannot be simply understood as the superposition of tensile and torsional fracture mechanisms. As can be seen from Figure 15c, under the multiaxial non-proportional loading conditions in this study, the fracture surface is still dominated by fatigue striations, but scratch marks can still be seen in Figure 15f. According to Figure 15i, the activation of slip systems leads to the formation of cellular structures, which have the greatest resistance to deformation among the substructures. This will strongly inhibit subsequent dislocation movement and significantly increase the material’s hardening rate, thus leading to an additional hardening effect. As shown in Figure 15a,d,g, under pure uniaxial tension, the instantaneous fracture zone exhibits typical equiaxed dimples formed by normal stress. Specimens only subjected to torsional loading, as shown in Figure 15b,e,h, fail under shear stress, resulting in twisted dimples with elliptical and tear-shaped elongations. Under multiaxial loading, as shown in Figure 15c,f,i, the instantaneous fracture zone still predominantly features equiaxed dimples since the specimens are ultimately pulled apart by normal stress. The presence of a phase difference reduces the number of dimples, as some small and shallow dimples are worn away by shear stress, making the surrounding areas of the remaining dimples smoother.

**Figure 16:**
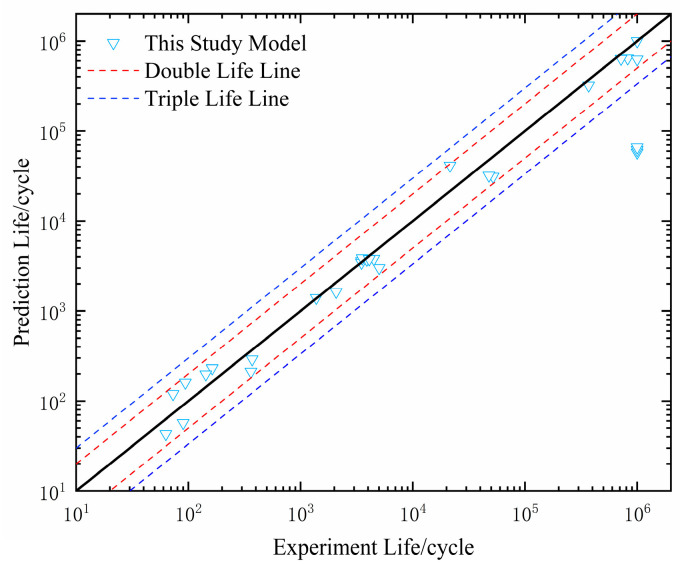
Comparison of predicted results by the present model with experimental results.

Finally, to validate the accuracy of the lifetime prediction model considering additional hardening effects and stress gradients, an isochronous lifetime diagram was established by comparing the experimental and simulation results, as shown in Figure 16. It was found that when the lifetime is in the low-cycle fatigue range, 100% of the prediction results are within the 2× life band, while when the lifetime is in the high-cycle range, the prediction accuracy significantly decreases (with 10^5^ as the boundary, meaning values above 10^5^ are classified as high-cycle values). 

**Figure 17:**
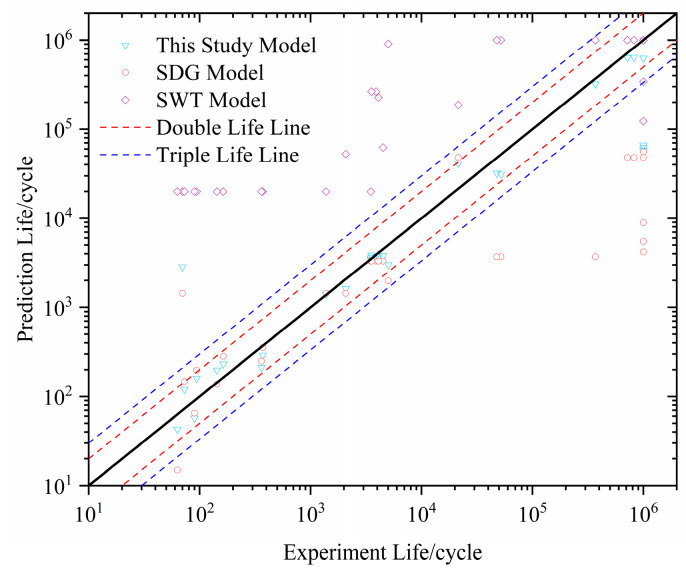
Comparison of predictions from three different models.

To validate the feasibility and precision of Equation (16) for assessing notched components, the life prediction conditions of the present model, the SWT model, and the multiaxial fatigue damage model proposed by Shang Deguang were compared. As can be seen from Figure 17, the present model has good prediction accuracy, with the prediction results lying within the 2× life band for all stages (except for 10^6^). For the Shang Deguang model, 65.7% of the results are within the 2× life band, while the SWT model has only 8% of the results within the 2× life band, indicating a larger error.

The lifetime of the material is evaluated using probabilistic analysis error, as shown in Equation (18). This approach allows for a quantitative comparison and analysis of the prediction capabilities of the three models:

(18) $$P_{error}=\mathrm{lg}N_{p}-\mathrm{lg}N_{f}$$

**Figure 18:**
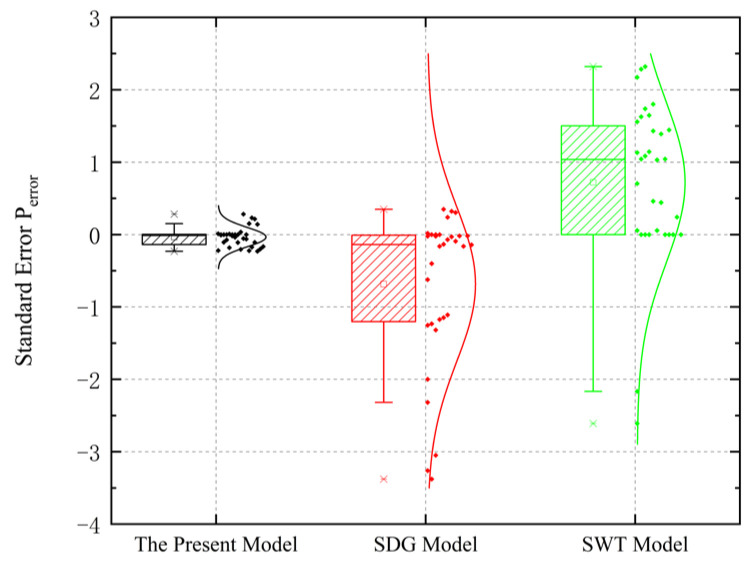
Box plots of prediction errors for three models.

The selected models are evaluated using box plots combined with normal distribution curves, where positive and negative values of the P error reveal whether the prediction results are greater than or less than the actual values, respectively. The height of the box reflects the standard deviation, and the curve represents the normal distribution. As can be seen from Figure 18, the average value for the present model is close to 0, with a small standard deviation, and the normal distribution curve appears “tall and thin.” The standard deviations of the Shang Deguang model and the SWT model are smaller under proportional loading than under non-proportional loading, their normal distribution curves appear “short and fat,” and both models have larger prediction errors and dispersion than the present model. It can be concluded from the above analysis that the present model has higher precision.

## 7. Summary

1. This paper analyzes the uniaxial nonlinear fatigue damage prediction model and determines the control parameter $\frac{\Delta \sigma }{2}$ of the uniaxial fatigue life prediction model based on damage mechanics, and an equivalent stress gradient coefficient $Y_{eq}$ is introduced to construct a new damage parameter.
2. This paper analyzes the loading conditions of gas turbine compressor blades, selects notched specimens as test pieces based on the unique loading characteristics of the blades, and designs experimental schemes based on different loading paths to validate the model presented in this paper. At the same time, it provides a basis for predicting the fatigue life of real blades under actual service loads.
3. The stress distribution of the notched specimen was calculated using finite element software, and the critical plane angle was determined. A comparison was made with the actual experimental fracture surface. It was found that, except for the conditions with a relatively large torque, the results were satisfactory in other cases. This validation confirmed that the damage parameter of the SWT model can reflect the damage mode of the blades and is more suitable for tensile crack models.
4. A life prediction model considering the additional strengthening effect and stress gradient was established based on the non-proportionality factor defined by Borodii. It was compared with the SWT model and the Shang Deguang model, and the results show that the present model is almost entirely within the 2× life band, indicating more accurate prediction results.
5. Analysis of the fracture surfaces of specimens subjected to uniaxial tension, torsion, and multiaxial loading reveals that the additional hardening effect of TC4 material under multiaxial loading is due to the activation of slip systems, which form cellular structures and produce significant resistance to deformation.

## Figures and Tables

**Table 1:** Damage parameters of TC4 alloy.

$M_{0}$	β	$\sigma_{-1}$ (MPa)	$a_{1}$	$\sigma_{b}$ (MPa)	$b^{\prime }$
2.9231 × 10^9^	0.46	346	0.4838	873	9.2 × 10^−4^
E (MPa)	$\sigma_{f}^{\prime }$ (MPa)	$\varepsilon_{f}^{\prime }$	b	c	
108.4	1136.9	0.579	−0.049	−0.679	

**Table 2:** The chemical composition of TC4.

Component	Alloying Element				Impurities				
	Al	V	Ti	Fe	C	O	H	N	Other Elements
Content/%	6.17	4.2	Margin	0.11	0.03	0.14	0.0001	0.01	<0.3

**Table 3:** Chaboche model: hardening parameters.

Material parameters	$c_{1}$	$\gamma_{1}$	$c_{2}$	$\gamma_{2}$	$c_{3}$	$\gamma_{3}$
Numerical value	129,487	1301	28,071	420	22,027	0

**Table 4:** Fatigue life data.

Loading Path	F (kN)	T (N*m)	θc	φc	$l_{np}$	$Y_{eq}$	$\bar{N_{f}}$	Prediction Results
1-I	5.788	24.11	117	33	1	1.786	70	2850
2-I	5.265	16.27	58	27	1	1.874	3500	3587
3-I	7.593	8.94	62	21	1	1.689	5020	3016
4-I	6.09	5.47	63	19	1	1.633	>10^6^	>10^6^
5-I	6.45	5.37	64	18	1	1.614	>10^6^	>10^6^
6-I	5.5	4.86	64	19	1	1.920	>10^6^	>10^6^
7-II	5.788	24.11	112	36	1.177	1.852	1380	1400
8-II	5.265	16.27	113	35	1.177	1.840	3500	3450
9-III	5.788	24.11	61	33	1.061	1.892	2080	1633
10-III	5.265	16.27	118	145	1.061	1.929	4500	3790
11-IV-0.7	5.265	16.27	112	35	1.177	1.776	>10^6^	>10^6^
12-IV-0.7	5.788	24.11	112	35	1.177	1.799	>10^6^	>10^6^
13-IV-0.1	5.265	16.27	113	35	1.177	1.780	>10^6^	57,838
14-IV-0.1	5.788	24.11	131	117	1.177	1.801	>10^6^	61,607
15-V-0.7	5.265	16.27	58	36	1.061	2.015	>10^6^	>10^6^
16-V-0.1	5.265	16.27	45	121	1.061	2.108	>10^6^	66,012
17-VI-30°	5.265	16.27	132	119	1.143	2.017	3900	3736
18-VI-45°	5.265	16.27	45	129	1.177	2.208	3550	3848
19-VI-60°	5.265	16.27	45	128	1.20	2.157	4100	3770
20-VII-30°	5.265	16.27	131	117	1.143	2.133	824,000	640,570
21-VII-45°	5.265	16.27	132	118	1.177	2.078	>10^6^	623,740
22-VII-60°	5.265	16.27	133	125	1.20	2.185	718,000	633,050
23-VII-45°	5.265	24.11	133	122	1.177	2.150	21,500	41,244
24-VII-45°	5.265	8.94	49	55	1.177	2.064	>10^6^	>10^6^
25-I	5.4	9.72	52	61	1	1.786	370,000	320,580
26-II	5.4	9.72	51	58	1.177	2.110	52,000	31,125
27-III°	5.4	9.72	134	127	1.061	2.183	47,700	31,945
28-IX	9.4	46.29	56	30	1.23	1.493	371	290
29-IX	9.767	47.25	64	37	1.23	1.497	163	231
30-IX	10.06	49.55	62	36	1.23	1.460	94	160
31-IX	10.31	50.77	60	35	1.23	1.456	73	120
32-X	9.4	50.77	50	115	1.167	1.523	361	212
33-X	9.92	53.58	51	116	1.167	1.320	143	198
34-X	10.31	55.67	51	115	1.167	1.594	90	57
35-X	10.62	57.34	51	73	1.167	1.407	63	43

## Data Availability

The original contributions presented in this study are included in the article. Further inquiries can be directed to the corresponding authors.
